# Coordinating Carbon Metabolism and Cell Cycle of *Chlamydomonas* *reinhardtii* with Light Strategies under Nitrogen Recovery

**DOI:** 10.3390/microorganisms9122480

**Published:** 2021-11-30

**Authors:** Yuanyuan Ren, Han Sun, Jinquan Deng, Yue Zhang, Yuelian Li, Junchao Huang, Feng Chen

**Affiliations:** 1 Institute for Food and Bioresource Engineering, College of Engineering, Peking University, Beijing 100871, China; 1701111648@pku.edu.cn (Y.R.); sunhanias@163.com (H.S.); zhangyue_daisy@pku.edu.cn (Y.Z.); yuelianli@yeah.net (Y.L.); 2 Shenzhen Key Laboratory of Marine Microbiome Engineering, Institute for Advanced Study, Shenzhen University, Shenzhen 518060, China; 1900392002@email.szu.edu.cn; 3 Institute for Innovative Development of Food Industry, Shenzhen University, Shenzhen 518060, China

**Keywords:** nitrogen recovery, cell cycle, *Chlamydomonas reinhardtii*, light quality, carbon metabolism

## Abstract

Nutrient supplementation is common in microalgae cultivation to enhance the accumulation of biomass and biofunctional products, while the recovery mechanism from nutrient starvation is less investigated. In this study, the influence of remodeled carbon metabolism on cell cycle progression was explored by using different light wavelengths under N-repletion and N-recovery. The results suggested that blue light enhanced cell enlargement and red light promoted cell division under N-repletion. On the contrary, blue light promoted cell division by stimulating cell cycle progression under N-recovery. This interesting phenomenon was ascribed to different carbon metabolisms under N-repletion and N-recovery. Blue light promoted the recovery of photosystem II and redirected carbon skeletons into proteins under N-recovery, which potentially accelerated cell recovery and cell cycle progression. Although red light also facilitated the recovery of photosystem II, it mitigated the degradation of polysaccharide and then arrested almost all the cells in the G1 phase. By converting light wavelengths at the 12 h of N-recovery with blue light, red and white lights were proved to increase biomass concentration better than continuous blue light. These results revealed different mechanisms of cell metabolism of *Chlamydomonas reinhardtii* during N-recovery and could be applied to enhance cell vitality of microalgae from nutrient starvation and boost biomass production.

## 1. Introduction

Microalgae excel in adapting to various culturing conditions. The advantages of microalgae in alleviating environmental issues and providing clean biofuels establish their competence in environmental and renewable energy arenas [1]. Unfortunately, their commercialized applications are challenged by the potential conflict between the cost of culturing processes and the revenue of bioproducts. The coordination of process-compatible products for specific market scenarios is considered as a promising orientation to improve the economic viability of microalgae production, which requires a comprehensive understanding of the relationship between microalgal cell growth and its biomass compositions [2,3]. As the cultivation process goes, nutrient concentration cannot afford subsequent cell growth; thus, microalgae have to slow down growth and convert nutrients into storage forms, such as starch and lipid. Only after nutrients are re-provided to the starving environment would the progression of the cell cycle be activated to advocate cell growth and, subsequently, cell division.

Nitrogen is an essential nutrient for microalgal growth, and nitrogen starvation (N-starvation) is commonly regarded as an effective stressing method to orient carbon partitioning among process-compatible products [4]. As nitrate concentration decreases, carbon partitioning would firstly be oriented to starch as a stored substance and to lipid as a secondary metabolite when nitrogen sources are exhausted in the culture [4].

Though studies about N-starvation on the accumulation of microalgal high-value products are numerous, few focus on the cell cycle during the transition process from N-starvation to nitrogen repletion (N-repletion), defined as N-recovery, which is common in fed-batch culture and the activation of microalgal seed cells. In such a situation, intermediate metabolisms are remodeled and cell division is discontinued [5], which affect the benefit of ultimately targeted bioactive compounds. Such interactions suggest that the productivity of process-compatible products is linked to the cell cycle to some extent.

The common cell cycle consists of the prolonged growth (G1) phase, DNA synthesis (S phase), mitosis (M phase), and cytokinesis. Normally, cell division is aroused by the mother cell size at a certain degree, as the cellular turgor pressure reaches the yield threshold for cell expansion. The model species *Chlamydomonas reinhardtii* is usually divided by a multiple fission mechanism that allows a single mother cell to produce 2 to 32 daughter cells [6]. A recent study revealed that the disruption of the cell cycle led to the remodeling of intermediate metabolisms, which consequently changed cell composition [7]. However, cell cycle progression under N-recovery is rarely reported.

To harness the cell cycle for maximum biomass value, it remains to be exploited whether the remodeling of intermediate metabolisms conversely influences cycle progression and how exactly abiotic factors work [8]. Environmental turbulence, such as light intensity and temperature, has been proved to affect the cell composition of microalgae at the metabolic level [9] and regulate the cell cycle by differential mechanisms [10,11]. Furthermore, several studies revealed that light wavelengths interacted with cell division and effectively reallocated carbon partitioning [12]. As the recovery of cell compositions for division is essential for the progression of the cell cycle, the interference of the recovering metabolic process by supplementing essential nutrients might lead to changes in the cycle [13,14]. For example, red light is valid for carbohydrate accumulation, while blue light is beneficial for protein synthesis for numerous algal species [15]. Therefore, light wavelength-induced changes in the cell cycle may also be linked to the remodeling of intermediate metabolisms during N-recovery. Though a number of growth models have been established to optimize cultivation parameters [16,17], few correlate with central carbon metabolism and cell cycle manipulation by applying different light wavelengths.

The objective of the current study was to reveal the different mechanisms of the cell metabolism of *Chlamydomonas reinhardtii* during N-recovery. The contribution of carbon metabolism to the cell cycle and biomass accumulation was analyzed under white, blue, and red light-emitting diode (LED) lights by measuring the photosystem performances, carbon partitioning, and central carbon metabolites of *C. reinhardtii*. To deeply understand the effects of light quality on cell behaviors during N-recovery, a kinetic model was designed to explore the metabolic behaviors of proteins and carbohydrates. Finally, a strategy was proposed to accelerate the recovery of green alga from N-starvation to enhance biomass production.

## 2. Materials and Methods

### 2.1. Microalgae Strain and Medium

*Chlamydomonas reinhardtii* CW15+ was obtained from the *Chlamydomonas* Resource Center (http://www.chlamycollection.org/, accessed on 19 October 2019). The seed cells were cultivated at 23 °C in Tris-acetate-phosphate (TAP) medium containing 7.5 mM NH_4_Cl under continuous illumination of 30 μmol photons m^−2^ s^−1^ [12]. To culture microalgae in N-starvation, cells were collected in the stationary growth phase, washed with N-free TAP medium (TAP-N, TAP medium without NH_4_Cl), and resuspended in TAP-N. The cells in TAP and TAP-N at the logarithmic growth phase were used as the seed cells for the following N-repletion and N-recovery cultivation, respectively.

### 2.2. Culture Conditions and Light Source

Afterward, 10 mL of two types of seed cells was inoculated separately in 250 mL Erlenmeyer flasks containing 100 mL TAP medium at 23 °C for 4 days under different light sources at 30 μmol photons m^−2^ s^−1^ in N-repletion and N-recovery conditions. The group with seed cells from TAP-N medium was defined as N-recovery, and the group with seed cells from TAP medium was defined as N-repletion. As for the light source, the equipment and light characteristics are described as follows: The properties of different LEDs are listed as the peak emission spectrum and its full width at half maxima (FWHM). LED strips (SuperFlux LED, Lumitronix LED-Technik GMBH, Hechingen, Germany) emitting blue (peak: 465 nm, FWHM: 18.5 nm) and red lights (peak: 635 nm, FWHM: 17 nm) were estimated for effects on microalgal growth, while white light was set as control. Three cultures were prepared for each culturing condition.

### 2.3. Determination of Microalgal Biomass

To determine the biomass concentration, 3 mL of the culture was collected by centrifugation at 6000× *g* for 5 min at room temperature. The supernatant was discarded, and the centrifugation procedure was repeated twice with distilled water to remove residual nutrients. The cell pellets were collected and filtrated on dry pre-weighed filter papers. The filter papers were then dehydrated in vacuum at 80 °C overnight. Finally, the filter papers were transferred to the desiccator to cool down, and the biomass concentrations were measured and calculated by the weight difference.

### 2.4. Quantification of Lipid, Carbohydrate, and Protein Contents

Microalgal lipid, protein, and carbohydrate contents were determined as previously described with slight modifications [18]. For quantifying lipid content, 20 mg of lyophilized cells was extracted with a mixture of chloroform, methanol, and water (*v/v/v*, 8:4:3). After the mixture was stratified, the organic phase was washed with 5% (*w/v*) sodium chloride and evaporated under nitrogen gas and dried at 60 °C in a vacuum oven to a constant weight. An analytical balance was then applied to measure the weight of lipid.

A total of 2 mL of the culture was centrifuged at 5000× *g* for 5 min and washed twice with distilled water. Then, the microalgal pellet was collected for determination of protein and carbohydrate contents. To quantify proteins, the pellet was hydrolyzed in 1 M NaOH at 80 °C for 10 min. Then, distilled water was added, and the mixture was centrifuged at 13,000× *g* for 30 min at 4 °C. This extraction procedure was repeated twice, and the supernatants were merged to quantify protein concentration using Protein BCA assay kit (Bio-Rad #5000002, Hercules, CA, USA).

To quantify carbohydrates, the microalgal pellet was mixed well with CH_3_COOH and then kept at 80 °C for 20 min. Then, acetone was added, and the mixture was centrifuged at 5000× *g* at room temperature for 10 min. The supernatant was discarded, and the pellet was mixed with 4 M trifluoroacetic acid and incubated at 100 °C for 4 h. Then, it was cooled down to room temperature and centrifuged at 13,000× *g* for 5 min. The supernatant was collected, and the phenol–sulfuric acid method was used to determine the total carbohydrate content.

### 2.5. Cell Cycle Analysis by Flow Cytometry

In our study, flow cytometry was applied to analyze the cell cycle of *C. reinhardtii*. The cells were cultured with white, blue, and red LED lights in N-recovery for 12 h. After the cultivation, the cells were collected by centrifugation at 1000× *g* for 5 min. The supernatant was discarded, and the pellets were washed twice with 1 mL of PBS. The cells were then re-suspended with 4 mL of pre-cooled ethanol (95%, *v/v*) and vortexed at low speed. To fix microalgal cells, the mixture was stored at 4 °C overnight. The cells were collected after centrifugation at 1000× *g* for 5 min. The pellets were collected and washed twice with 5 mL of PBS. Then, a cell cycle and apoptosis detection kit (CW2575S, CWBIO, Beijing, China) was applied to measure the cell cycle.

### 2.6. Analysis of Pigment Contents

The pigment contents were determined using the photometric method [19]. The cells were collected by centrifugation at 13,000× *g* for 5 min. The pellet was crushed with pre-cooled tissue crusher and redissolved in 99.9% methanol and vortexed sufficiently. This procedure was carried out in the dark. The extracts were centrifuged at 13,000× *g* for 10 min at 4 °C, and the supernatant was collected to measure the absorbance values at 470, 652.4, and 665.2 nm with a spectrophotometer. Pigment concentrations were calculated using the following equations:

(1) $$\mathrm{Chl}\;a\;\left(\mu g/\mathrm{mL}\right)=16.72\left(A_{665.2}\right)-9.16\left(A_{652.4}\right)$$

(2) $$\mathrm{Chl}\;b\;\left(\mu g/\mathrm{mL}\right)=34.09\left(A_{652.4}\right)-15.28\left(A_{665.2}\right)$$

(3) $$\mathrm{Carotenoids}\;\left(\mu g/\mathrm{mL}\right)=\frac{1000\left(A_{470}\right)-1.63\left(\mathrm{Chl}\;a\right)-104.9\left(\mathrm{Chl}\;b\right)}{221}$$

### 2.7. Measurement of Quantum Yield and Electron Transport Rate (ETR)

To analyze the characteristics of photosystem II, the maximum quantum yield (*F_v_/F_m_*), photosynthetic yield (YII), non-photochemical quenching (NPQ), and electron transport rate (ETR) were measured using pulse-amplitude-modulated fluorometry (Walz, Effeltrich, Germany). Before analysis, the microalgal cells were diluted to a similar absorbance at 680 nm with PBS and retained in the dark for 20 min for dark adaption. The minimum level of fluorescence (*F_0_*) and the maximum level of fluorescence (*F_m_*) were used to calculate *F_v_/F_m_* according to the following equation:

(4) $$F_{v}/F_{m}=\left(F_{m}-F_{0}\right)/F_{m}$$

### 2.8. Analysis of Total Fatty Acid (TFA)

The quantification of fatty acid methyl esters (FAMEs) was according to a previous study with a few modifications [20]. Accurately weighted 20 mg of lyophilized cells was mixed with 2 mL H_2_SO_4_ (0.1% *v/v* in methanol with 0.05% 2,6-Di-tert-butyl-4-methylphenol) and 1 mL toluene. Then, 0.5 mL of heptadecanoic acid (1 mg/mL in hexane) was added as the internal standard and vortexed well. The mixture was kept at 50 °C overnight and mixed with 1 mL 0.75% NaHCO_3_ after it had cooled to room temperature. Then, 2 mL hexane was added and kept still until layer separation. The upper layer was collected and centrifuged at 12,000× *g* for 10 min before analysis. The FAMEs were analyzed using a gas chromatography–mass spectrometry (GC-MS) (Shimadzu, Kyoto, Japan) equipped with an Agilent DB-WAX 122-7032 column (30 m × 0.25 mm × 0.25 μm) (Agilent, CA, USA). The initial oven temperature was set to 45 °C, subsequently raised to 150 °C at 15 °C/min and then to 240 °C at 6 °C/min, and finally kept at 240 °C for 6 min.

### 2.9. Metabolite Extraction and Analysis

The microalgal cells were centrifuged at 5000× *g* for 5 min at 4 °C. Then, pre-chilled methanol solution (80%, *v/v*, methanol in water) was added into the pellet for 3 min to quench microalgal metabolism. Afterward, cells were collected after centrifugation at 5000× *g* for 5 min at 4 °C, and the pellets were resuspended in pre-chilled methanol solution (80%) and frozen with liquid nitrogen. After 20 min incubation at −20 °C, the supernatant was collected after centrifugation for 5 min at 4 °C. The resuspension was then repeated twice. The merged supernatants were vacuum dried, and the residuals were dissolved in 80% methanol and analyzed using UPLC-MS/MS (Waters, Milford, MA, USA). The tandem system was equipped with an HSS T3 column (C18, 2.1 mm × 100 mm, 1.8 μm particle size) with a flow rate set as 0.4 mL/min, and the linear gradient procedure was set as follows: (a) 100% solvent B (H_2_O/HCOOH/0.2 M NH_4_Ac in water, 94.9:0.1:5, *v/v/v*) to 60% solvent A (C_2_H_3_N/HCOOH/0.2 M NH_4_Ac in water, 94.9:0.1:5, *v/v/v*); (b) 100% solvent A, held for 1 min; and (c) 100% solvent B and held for 4 min. The system was operated in the positive and negative ESI modes, and quantification was performed using the multiple reaction monitoring (MRM) mode. The optimal operating conditions were set according to our previous study [4].

### 2.10. Kinetic Models of Carbon Partitioning

Among the carbon sinks of microalgae, carbohydrates and proteins were mainly involved in cell division, whose accumulation and conversion may be closely related to cell cycles. Generally, nutrients in the medium were absorbed and transported into microalgal cells and then oriented into two main branches as carbohydrates and proteins. Thereafter, as the cells grew, some proteins would turn to degradation and partially flow to carbohydrates. Meanwhile, the conversion of carbohydrates also existed. Thus, assuming that nitrogen was the only limiting nutrient in cell growth, kinetic models of *C. reinhardtii* were built to predict behaviors of carbon partitioning under different wavelengths according to an earlier study as follows [21]:

(5) $$\frac{dQ_{C}}{dt}=Y_{CS}\frac{dS}{dt}-\alpha_{C}Q_{C}$$

(6) $$\frac{dQ_{P}}{dt}=Y_{PS}\frac{dS}{dt}-D_{P}Q_{P}$$

where *t* (h) is the cultivation time; *Q_C_* (g L^−1^) and *Q_P_* (g L^−1^) denote the concentrations of intercellular carbohydrates and proteins, respectively; *Y_CS_* and *Y_PS_* denote carbohydrate and protein yields on nutrient concentration *S* (g L^−1^). *α_C_* (h^−1^) and *D_P_* (h^−1^) denote the conversion rates of carbohydrates and proteins, respectively. Supposing the concentrations of carbohydrates and proteins were proportional to the biomass (g L^−1^), equations were rewritten as the following:

(7) $$\frac{dQ_{C}}{dt}=Y_{CX}\mu X_{0}exp\left(\mu t\right)-\alpha_{C}Q_{C0}exp\left(\mu_{C}t\right)$$

(8) $$\frac{dQ_{P}}{dt}=Y_{PX}\mu X_{0}exp\left(\mu t\right)-D_{P}Q_{P0}exp\left(\mu_{P}t\right)$$

where *μ* (h^−1^), *μ_C_* (h^−1^), and *μ_P_* (h^−1^) denote specific rates of cell growth and carbohydrate and protein concentrations. *Y_CX_* (g g^−1^) and *Y_PX_* (g g^−1^) are carbohydrate and protein yields on biomass concentration. In these equations, the initial biomass concentration was set as X_0_ (g L^−1^). All the models above presupposed that cultivation conditions were maintained as invariants and that the extracellular metabolites during the exponential growth phase would not have any impacts on cell growth.

### 2.11. Statistical Analysis

All the experiments above were conducted in at least three individual replicates to guarantee reproducibility. Statistical analysis was carried out using IBM SPSS 18.0 software and Origin 8.5. A one-way analysis of variance (ANOVA) was applied for the determination of the significant differences from the control groups for each experimental condition separately, and *p* < 0.05 was considered as statistically significant. All the data are presented in the form as means value (*n* = 3) ± the standard deviation.

## 3. Results and Discussion

### 3.1. Effects of Light Wavelengths on Cell Growth and Photosystem under N-Repletion

**Figure 1:**
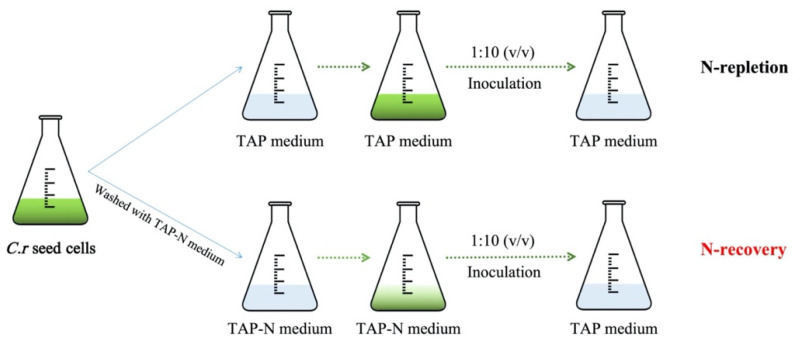
Schemes of two experimental conditions of *C. reinhardtii* as N-repletion and N-recovery.

Light sources, with a narrow spectral output overlapping the photosynthetic absorption spectrum available for microalgae, are needed to increase the conversion efficiency of light energy, potentially promoting biomass accumulation and bioproduct biosynthesis [11]. Therefore, LED lights of white, blue, and red were chosen to explore the effects on the cell growth of *C. reinhardtii* in both N-repletion and N-recovery (Figure 1).

**Figure 2:**
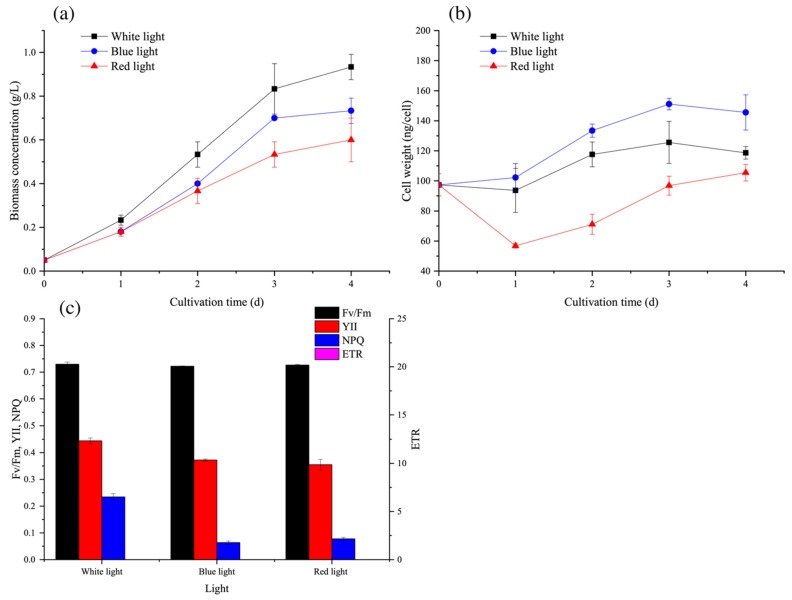
Effect of light wavelength on (**a**) biomass concentration; (**b**) cell weight; and (**c**) photosystem II on the 4th day of *C. reinhardtii* under N-repletion. Data are presented in the form of mean ± the standard deviation (*n* = 3).

As shown in Figure 2a, the biomass accumulation under N-repletion under white light was 21.4% and 35.7% higher than that under blue and red lights, respectively, which suggested that other light wavelengths in white light were also beneficial for cell growth. Their absence in red and blue lights would limit the biomass concentration. Moreover, blue and red lights also exhibited different effects on cell enlargement and cell division. Under N-repletion, blue light increased cell weight but with a lower cell number, while red light treatment gave rise to a higher cell number but a lower cell weight as compared with white and blue lights (Figure 2b and Figure S1). This may suggest that red light was more preferable than blue light for cell division under N-repletion.

As for the effects of light quality on photosystems (Figure 2c), the YII, ETR, and NPQ values under white light were all significantly higher than those under blue and red lights (*p* < 0.05), suggesting that the working efficiency of the photosystem was actively stronger under white light. Meanwhile, there were no significant differences in *F_v_/F_m_* values, indicating no inhibition on photosynthesis under blue and red lights.

### 3.2. Effects of Light Wavelengths on Cell Growth and Cell Cycle under N-Recovery

**Figure 3:**
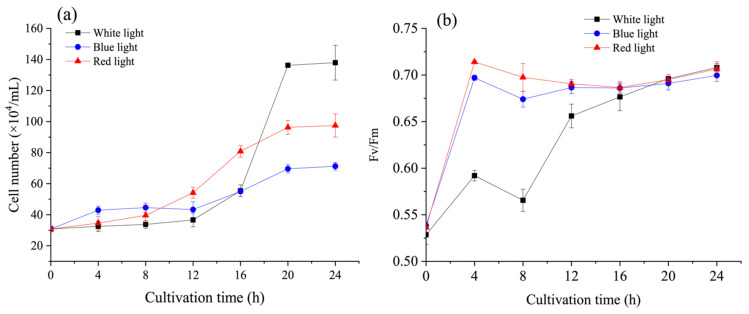
Effect of light wavelength on (**a**) cell number and (**b**) *F_v_/F_m_* of *C. reinhardtii* under N-recovery. Data are presented in the form of mean ± the standard deviation (*n* = 3).

Under N-recovery, the effects of different light on cell enlargement and division were considerably distinct compared with those under N-repletion (*p* < 0.05). Under N-recovery, white light exhibited a greater potential in promoting cell growth than red and blue lights, which was in accordance with the results under N-repletion (Figure 3a, Figures S2 and S3). It is worth noting that during the initial 4 h of treatment, blue light increased cell number with a lower cell weight than the other two lights (Figure 3a and Figure S4). Interestingly, although blue light was acknowledged to effectively upregulate the transcription factors of cell enlargement [22], it was demonstrated in this study to promote cell division under N-recovery.

**Figure 4:**
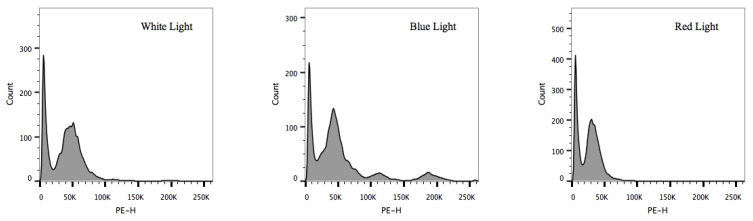
Cell cycle distribution of microalgal cells under white light, blue light, and red light under N-recovery.

Since these physiological changes were linked to the cell cycle, cycle phases were measured at the 12 h of cultivation under N-recovery. As shown in Figure 4, cells in the G1 phase occupied the majority of the cell cycle, indicating that the cell cycle was arrested under N-starvation. After moving to N-recovery for 12 h, a relatively small number of cells under white light were pushed forward into S and G2 phases, which suggested that the cells were partly activated to divide. Interestingly, blue light induced more cells into S and G2 phases than white light, and cells in the M phase could also be observed, while red light kept blocking most of the cells from entering the S and G2 phases. These results indicated that blue light was preferable for cell division under N-recovery.

### 3.3. Photosynthetic Characteristics and Pigments of C. reinhardtii under N-Recovery

We first assumed that the unusual cell cycle distribution of microalgal cells under different light wavelengths might be ascribed to the different recovery mechanism of the photosystem, which is limited under N-starvation. As shown in Figure 3b, the light wavelengths influenced *F_v_/F_m_* during the recovery time of 24 h. Both red and blue lights promoted the recovery of Fv/Fm at the initial treatment of 12 h, especially at the first 4 h (Figure 3b). The values of *F_v_/F_m_* were 0.5920 ± 0.0057 (white), 0.6970 ± 0.0028 (blue), and 0.7140 ± 0.0014 (red) at 4 h, while those at 12 h were 0.6560 ± 0.0127 (white), 0.6865 ± 0.0064 (blue), and 0.6905 ± 0.0050 (red), respectively. Since *F_v_/F_m_* is considered as a stress indicator, its increase suggested that cell photosynthesis was enhanced when cells were exposed to blue and red lights at the same light intensity [4]. There were no significant differences in ETR values under different light wavelengths (Figure S5). In addition, the ratio of Chl *a* to Chl *b* was recovered toward a fixed value of 2:1 during the 24 h under white (2.80:1), blue (2.43:1), and red (2.51:1) lights (Figure S6). Specifically, blue and red lights promoted this recovery process by increasing Chl *b* contents at the initial 20 h. Since the light-harvesting antenna complexes contain Chl *a* and Chl *b* approximately with a ratio of 2:1, recovering and maintaining this ratio may be beneficial to the performance of the photosystem. The rapid generation of Chl *b* might result in a higher photosynthetic efficiency and induce the cells into cell cycle progression more rapidly upon blue or red light treatment as compared with white light.

However, although both blue and red lights could promote photosystem recovery at the initial 12 h as indicated by *F_v_/F_m_* and increased pigment content (Figure S7), their influences on the cell cycle were different. As pigment is not the determining factor of cell cycle progression and cell growth, the main carbon sinks were explored under different light wavelengths.

### 3.4. Carbon Partitioning of C. reinhardtii under Different Light Wavelengths under N-Recovery

Microalgae usually undergo a number of cell cycles and then present the final cell compositions during the batch culture of microalgae. Before cell division, protein content climbed to maximum and further converted to lipid and starch depending on environmental conditions [22]. Thus, microalgal cells at different cell cycle stages could lead to different biomass compositions, which has been proved by a previous study [5]. In our study, the effects of light wavelengths on cell growth during N-recovery were in contrast to the results of other studies that reported that blue light would boost cell enlargement and red light would favor cell division [23]. Thus, we hypothesized that during N-recovery, blue and red lights caused different carbon metabolism and further affected the cell cycle.

Proteins and carbohydrates are closely related to the cell cycle. Under optimal growth conditions, microalgal cells usually guide carbon molecules into the central metabolite, pyruvate, in the G1 phase, which is then preferentially allocated to protein synthesis through the TCA cycle [24]. Our previous study also suggested that carbohydrate content derived from protein content was large under stressing conditions, which would hinder cell division and growth [4]. Thus, the recovery of cell compositions from N-starvation is essential for recovering cell cycle progression.

**Figure 5:**
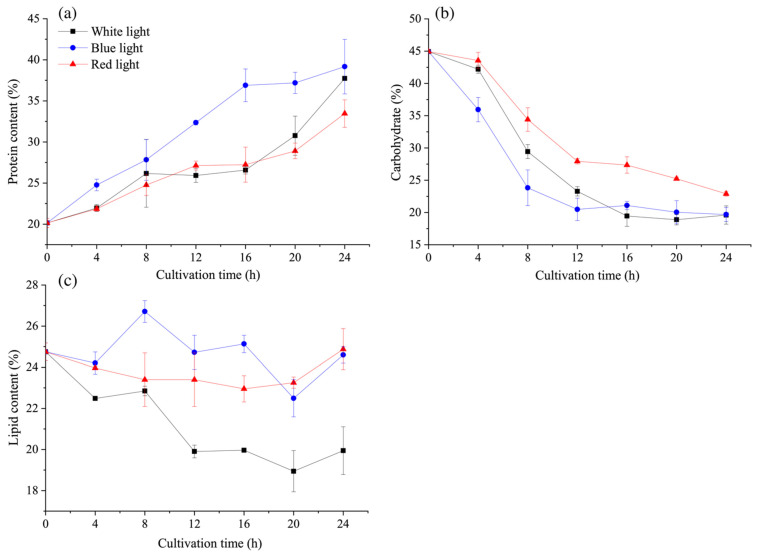
Effect of light wavelength on (**a**) protein content, (**b**) carbohydrate content, and (**c**) lipid content of *C. reinhardtii* under N-recovery. Data are presented in the form of mean ± the standard deviation (*n* = 3).

As shown in Figure 5, the results of the main carbon sinks under the N-recovery of *C. reinhardtii* exhibited significant differences under various light wavelengths (*p* < 0.05). In the initial 24 h, blue light exhibited as the best for protein production (Figure 5a), while red light significantly hindered the degradation of carbohydrates into essential carbon skeletons for protein synthesis (Figure 5b). It is interesting that both blue and red lights significantly delayed the degradation of lipids (Figure 5c), which indicated that the carbon skeletons for protein synthesis under blue light mainly came from carbohydrate degradation. During N-recovery, the increase in proteins under blue light contributed to cell division with more cells in S and G2 phases, while the storage of carbohydrates under red light limited cell cycle progression with low cell amounts in these two phases (Figure 4). As the precise carbon metabolism under different light wavelengths was more strong evidence for carbon sink changes, the main metabolites of central carbon metabolism were also quantified.

### 3.5. Metabolites in Central Carbon Metabolism of C. reinhardtii under N-Recovery

**Figure 6:**
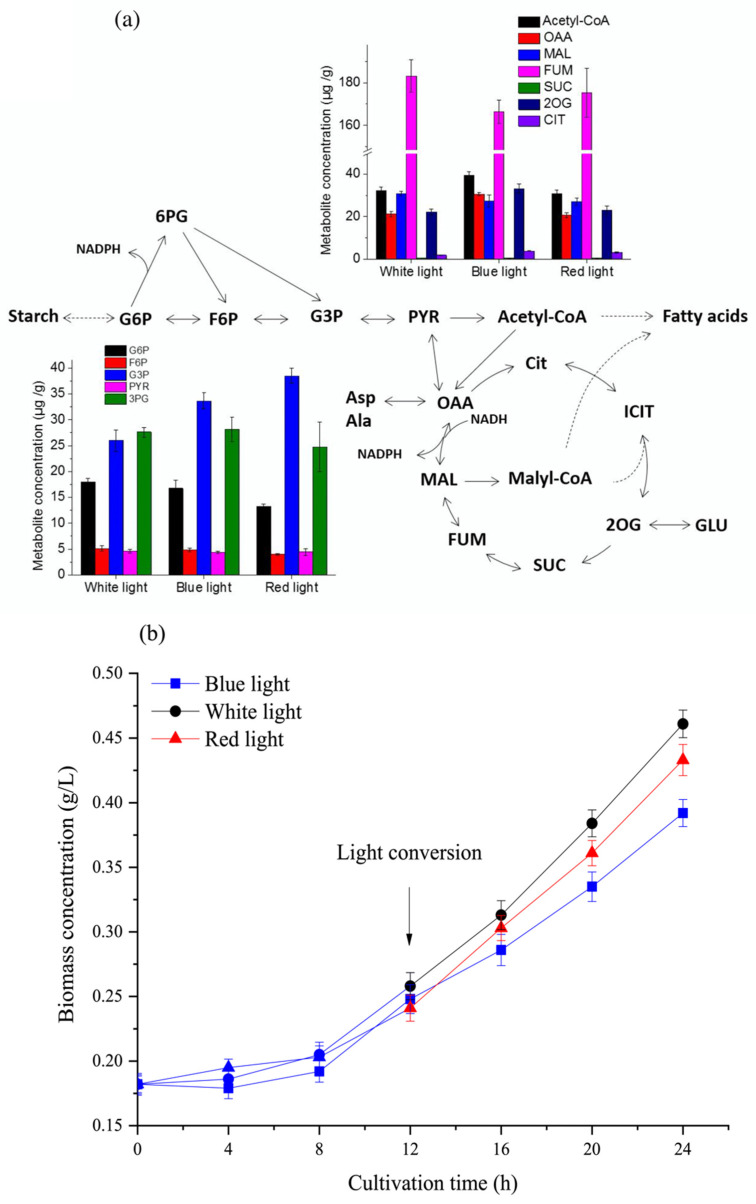
(**a**) Various metabolites of *C. reinhardtii* at different light wavelengths at 12 h of N-recovery. The carbon metabolites are the following: G6P, glucose-6-phosphate; F6P, fructose-6-phosphate; G3P, glyceraldehyde-3-phosphate; 3PG, 3-phosphoglycerate; PYR, pyruvate; OAA, oxaloacetate; MAL, malate; FUM, fumarate; SUC, succinate; 2OG, 2-oxoglutarate; and CIT, citrate. (**b**) Effect of light conversion at 12 h of cultivation on biomass concentration of *C. reinhardtii*. Data of metabolite concentration and biomass concentration are presented in the form of mean ± the standard deviation (*n* = 3).

As shown in Figure 6a, under blue light, the content of glyceraldehyde-3-phosphate (G3P) was significantly higher than that under white light (*p* < 0.05), suggesting that glycolysis was enhanced and supplied more available carbon skeletons. Acetyl-CoA was a key metabolic intermediate applying carbon skeletons, and its increase under blue light (Figure 6a and Table 1) may lead to an enhanced TCA cycle or lipid biosynthesis [10]. Among TCA cycle metabolites, the contents of oxaloacetate and 2-oxoglutarate, the precursors of aspartate and glutamate, increased under blue light. Combining these pieces of evidence with the results of carbon partitioning and cell cycle (Figure 4 and Figure 5) indicates that blue light could turn carbon skeletons from carbohydrates into proteins, which is essential for cell division.

As shown in Figure 5b, the carbohydrate contents were all decreased under N-recovery under the three different lights. Under red light, the G3P was the highest compared to that under blue and white lights, while the contents of glucose-6-phosphate (G6P) and fructose-6-phosphate (F6P) were relatively low (Figure 6a and Table 1). Considering carbohydrate degradation under red light was the lowest compared with the other two lights (Figure 5b), it was therefore believed that carbohydrate catabolism might be inhibited to some extent at the G3P point under red light. Since the Calvin–Benson–Bassham (CBB) cycle could also provide G3P, it was also hypothesized that red light might promote carbon fixation in photosynthesis [25], which was in accordance with the results from *Fv/Fm* (Figure 3b) and Chl contents (Figure S6). Hence, though red light had little influence on TCA cycle metabolites, it had great potential to pave CO_2_ into G3P and slow down carbohydrate degradation under N-recovery.

Additionally, decreased starch level after cell division could potentially limit lipid content since starch was the main carbon sink for lipid biosynthesis under stress conditions [15]. Light with different wavelengths could also lead to the changes in lipid content and total fatty acid profiles (Figure 5c and Table S1). As fatty acid profiles also act as the functional components in cell metabolism and growth [10], their changes could potentially affect cell cycle progression, and further study is needed to exploit their specific functions and synchronously manipulate TFA profiles for high-value bioproducts.

### 3.6. Kinetic Models of Carbon Sinks and Carbon Metabolism for Biomass Production

Under N-recovery, the proteins and carbohydrates of *C. reinhardtii* under different wavelengths were explored in the first 24 h and described with kinetic models. The models established before were well fitted (R^2^ > 0.95) with the experimental data, and the coefficients of the model after fitting are shown in Table 2. The values of *Y_CX_* were all negative under the three different lights, demonstrating that the microalgal cells consumed more intracellular carbohydrates than that accumulated at the beginning of N-recovery.

Under blue light, *Y_PX_* reached its highest value, indicating that the cells had the best capacity to convert nutrients into proteins essential for cell division. The positive value of *D_p_* suggested that the degradation of proteins also existed on the contrary. Additionally, the highest value of *α_C_* revealed that the degradation of carbohydrates was significantly propelled. Combining the information from the metabolite analysis presented in Figure 6a, it was obvious that increased amounts of OAA and 2-OG were in-depth proof for the conversion of carbohydrates to proteins, which could further propel the cell cycle from the G1 phase to division (Figure 4).

Under red light, the value of *α_C_* suggested that carbohydrate degradation was partially blocked, subsequently prone to arrest the cell cycle in the G1 phase [24]. Moreover, G6P and F6P were both significantly lower than those under other light wavelengths (*p* < 0.05) (Figure 6a). These results were consistent with the kinetic model. Moreover, *α_C_* could also be used as a reference parameter in cell cycle manipulation for further cultivation.

To sum up, the kinetic models of carbon storage and the key coefficients involved in the cell cycle (*Y_PX_, Y_CX_*, *D_P_,* and *α_C_*) could provide a promising perspective to predict and manipulate the cell cycle under N-recovery and further orient microalgal cultivation to a higher biomass concentration through cell cycle manipulation [21]. Therefore, we put forward a novel cultivation strategy to detect if a higher biomass of *C. reinhardtii* during N-recovery with light conversion could be achieved.

### 3.7. A Novel Mixed Light Strategy to Boost Biomass Production by C. reinhardtii under N-Recovery

According to the results described above, blue light accelerated the adaptation process under N-recovery, facilitating the faster adaptation from N-starvation at the beginning. However, it would not be a profitable strategy with prior cell division for higher biomass concentration in the long run. After recovery from N-starvation, the same condition as N-repletion, blue light would be prone to increase cell size under N-repletion (Figure 2b) when cell division was postponed. Thus, after the adaptation of microalgae to nutrient-sufficient conditions, red and white lights may be more preferable for cell cycle progression and a higher biomass concentration of the recovered cells (Figure 3a). This result could then lead to a new cultivation strategy. Several reported studies applied mixed lights to increase biomass concentration. For example, a mixed light of red and white resulted in more biomass concentration than white or red light alone [11]. Thus, different light wavelengths were applied after cell recovery and to explore the better choice.

Although various strategies could induce the accumulation of targeted biomolecules, most of them worked with extra expenses, such as inhibiting cell cycle progression, which largely limited biomass productivity [26]. Therefore, we designed a strategy focusing on increasing biomass concentration by remodeling carbon metabolisms to harness the cell cycle by tuning light wavelengths at chosen stages. As shown in Figure 6b, blue light was initially applied to accelerate cell recovery during the first 12 h, and then red and white lights were separately applied to induce cell growth during the subsequent 12 h. Under red and white lights, the biomass concentrations were, respectively, 10% and 17% higher than those under continuous blue light, ascribed to their efficient biosynthesis of metabolites for cell division when nutrition was sufficient. Though red light exhibited the greater potential in cell division under N-repletion (Figure S1), white light was more likely to boost biomass accumulation (7% higher than that of red light), suggesting that weak light wavelengths in white light might be beneficial for cell growth.

Taken together, remodeling carbon metabolisms could influence cell cycle progression, and a suitable cultivating strategy by switching light wavelengths at a chosen time point was promising to boost cell growth and biomass accumulation. Moreover, both coefficients of kinetic models and metabolite analysis should be simultaneously considered before designing a competent strategy.

Apart from light wavelengths, there are still other aspects to be optimized for a higher biomass. Macro-nutrient limitation could restrict cell cycle progression with the accumulation of carbohydrates, while proteins essential for cell division were in shortage [27]. Therefore, to maintain cell cycle progression, strategies to provide nutrient supplementation are promising, where fed-batch culture has been proved more efficient to accumulate biomass [28]. Our results of *C. reinhardtii* are consistent with those of previous studies on other microalgae species, such as *Nannochloropsis oceanica* [29], *Parachlorella kessleri* [30], and *Chromochloris zofingiensis* [31], regarding the notion that microalgae can recover from nitrogen starvation in 1–2 days. Furthermore, after achieving preferable biomass concentrations, stressing conditions, such as high light intensities and salinity treatment, could be used subsequently in multi-stage cultivation of microalgae, which remains to be further exploited.

## 4. Conclusions

Under N-recovery, the effects of blue and red lights on *C. reinhardtii* were different from those under N-repletion. During N-recovery, blue and red lights showed opposite effects on cell division and growth compared to regular patterns under N-repletion. Blue light stimulated the cell division of *C. reinhardtii* with sufficient proteins for quick recovery, while red and white lights contributed to cell growth. By tuning lights at 12 h, red and white lights increased the biomass by 10% and 17% compared with continuous blue light, respectively. Thus, regulating carbon metabolism could control the cell cycle, and it was significant to enhance the frequency of the cell cycle and lengthen the cell growth phase simultaneously for a higher biomass concentration and high-value bioproducts productivities. In conclusion, our study gathers the missing information regarding *C. reinhardtii* during N-recovery in terms of cell cycle and carbon metabolism and offers an innovative light strategy for accelerating the recovery progression of microalgae from nutrient-starving adversity and achieving a higher biomass concentration.

## Figures and Tables

**Table 1:** LC-MS results for various metabolites of *C. reinhardtii* at different light wavelengths at 12 h of N-recovery. Data in the table are presented in the form of means (*n* = 3) ± the standard deviation. The same letter (a, b, c) on each bar indicates that the difference is not significant (*p* > 0.05), and different letters indicate a significant difference (*p* < 0.05).

Metabolite	Metabolite Content (μg/g)		
	White Light	Blue Light	Red Light
Glycolysis and CBB cycle intermediates			
Glucose-6-phosphate	17.98 ± 0.67 ^b^	16.73 ± 1.62 ^b^	13.22 ± 0.47 ^a^
Fructose-6-phosphate	5.11 ± 0.53 ^b^	4.84 ± 0.34 ^b^	3.98 ± 0.19 ^a^
Glyceraldehyde-3-phosphate	25.98 ± 2.08 ^a^	33.59 ± 1.59 ^b^	38.45 ± 1.47 ^c^
Pyruvate	4.61 ± 0.38 ^a^	4.39 ± 0.21 ^a^	4.42 ± 0.62 ^a^
3-phosphoglycerate	27.59 ± 0.95 ^a^	28.13 ± 2.42 ^a^	24.75 ± 4.77 ^a^
Acetyl-CoA	32.15 ± 1.78 ^a^	39.48 ± 1.56 ^b^	30.72 ± 1.77 ^a^
TCA cycle intermediates			
Oxaloacetate	21.22 ± 1.29 ^a^	30.58 ± 0.86 ^b^	20.73 ± 1.12 ^a^
Malate	30.66 ± 1.16 ^a^	27.39 ± 2.89 ^a^	27.07 ± 1.78 ^a^
Fumarate	183.17 ± 7.58 ^a^	166.30 ± 5.49 ^a^	175.41 ± 11.53 ^a^
Succinate	0.58 ± 0.02 ^a^	0.48 ± 0.22 ^a^	0.49 ± 0.23 ^a^
2-oxoglutarate	22.14 ± 1.35 ^a^	33.09 ± 2.21 ^b^	23.05 ± 1.84 ^a^
Citrate	1.91 ± 0.17 ^a^	3.82 ± 0.10 ^c^	3.16 ± 0.27 ^b^

**Table 2:** Key coefficients of kinetic models for carbon storage in N-recovery. Data are presented in the form of mean ± the standard deviation (*n* = 3).

Coefficient	White Light	Blue Light	Red Light
*Y_PX, max_* (g g^−1^)	0.1608 ± 0.0145	0.2323 ± 0.0073	0.2013 ± 0.0074
*D_P_* (×10^−2^ h^−1^)	−0.0003 ± 0.0001	0.0001 ± 0.0000	−2.133 × 10^−7^ ± 0.0000
*Y_CX, max_* (g g^−1^)	−0.9289 ± 0.0401	−0.6269 ± 0.0430	−0.9013 ± 0.0593
*α_C_* (h^−1^)	0.0009 ± 0.0001	0.0071 ± 0.0011	0.0000 ± 0.0001

## Data Availability

Not applicable.

## Supplementary Materials

The following are available online at https://www.mdpi.com/article/10.3390/microorganisms9122480/s1, Figure S1: Effect of light wavelength on cell number on the 4th day of *C. reinhardtii* under N-repletion, Figure S2: Effect of light wavelength on biomass concentration and cell number of *C. reinhardtii* under N-repletion during the first 24 h, Figure S3: Effect of light wavelength on biomass concentration of *C. reinhardtii* under N-recovery, Figure S4: Effect of light wavelength on cell weight of *C. reinhardtii* under N-recovery, Figure S5: Effect of light wavelength on ETR *C. reinhardtii* under N-recovery, Figure S6: Effect of light wavelength on pigment profile of *C. reinhardtii* under N-recovery, Figure S7: Effect of light wavelength on pigment content of *C. reinhardtii* under N-recovery, Table S1: Fatty acid profiles in TFA of *C. reinhardtii* at different light wavelengths in N-recovery.
