# Supplementary material for: Coordinating Carbon Metabolism and Cell Cycle of Chlamydomonas reinhardtii with Light Strategies under Nitrogen Recovery
Source: Microorganisms. 2021 Nov 30;9(12):2480. doi: 10.3390/microorganisms9122480 (PMC8707240; doi:10.3390/microorganisms9122480)
Supplement: Supplementary file 1 [file microorganisms-09-02480-s001.zip › microorganisms-1445533-supplementary.pdf]

Supplementary data

Coordinating Carbon Metabolism and Cell Cycle of *Chlamydomonas reinhardtii* with Light Strategies under Nitrogen Recovery

Yuanyuan Ren [1,2,3], Han Sun [1,2,3], Jinquan Deng [2,3], Yue Zhang [1], Yuelian Li [1], Junchao Huang [2,3,*], Feng Chen [2,3,*]

1 Institute for Food and Bioresource Engineering, College of Engineering, Peking University, Beijing, 100871, China; 1701111648@pku.edu.cn (Y.R.); sunhanias@163.com (H.S.); zhangyue_daisy@pku.edu.cn (Y.Z.); yuelianli@yeah.net (Y.L.)

2 Shenzhen Key Laboratory of Marine Microbiome Engineering, Institute for Advanced Study, Shenzhen University, Shenzhen 518060, China; 1701111648@pku.edu.cn (Y.R.); sunhanias@163.com (H.S.); 2015020302@email.szu.edu.cn (J.D.); sfchen@szu.edu.cn (F.C.); huangjc65@szu.edu.cn (J.H.)

3 Institute for Innovative Development of Food Industry, Shenzhen University, Shenzhen 518060, China; 1701111648@pku.edu.cn (Y.R.); sunhanias@163.com (H.S.); 1900392002@email.szu.edu.cn (J.D.); sfchen@szu.edu.cn (F.C.); huangjc65@szu.edu.cn (J.H.)

* Correspondence: sfchen@szu.edu.cn (F.C.); huangjc65@szu.edu.cn (J.H.)

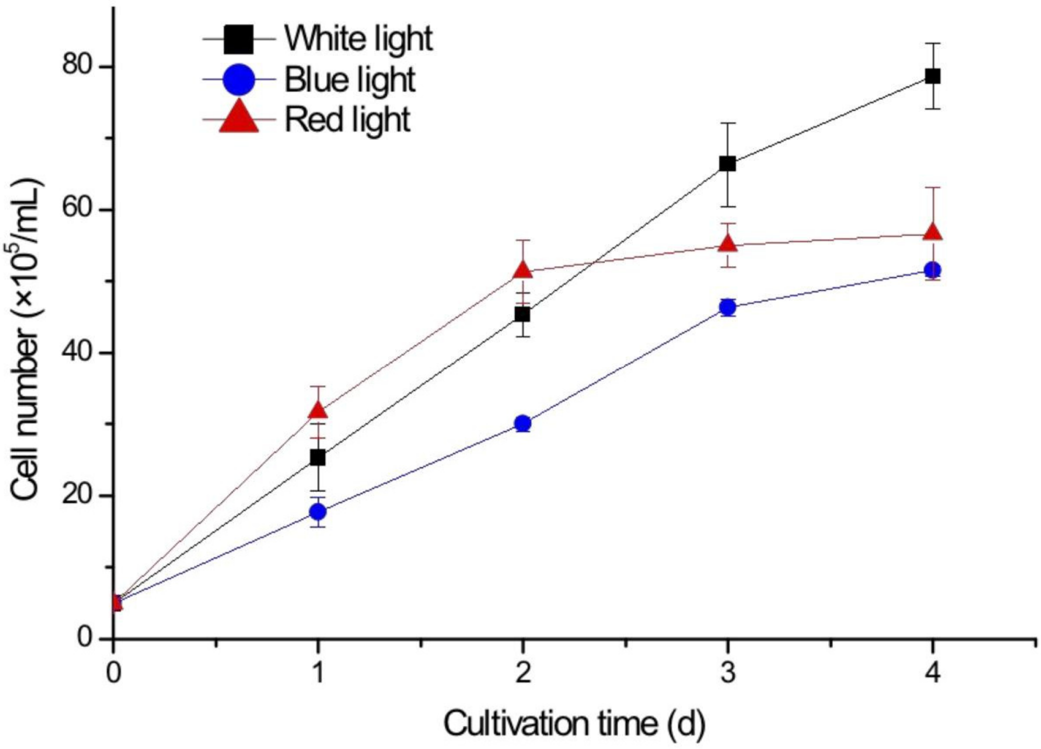

Supplemental Figure S1. Effect of light wavelength on cell number at the 4th day of *C. reinhardtii* under N-repletion. Data were presented in the form of mean ± the standard deviation (n = 3).

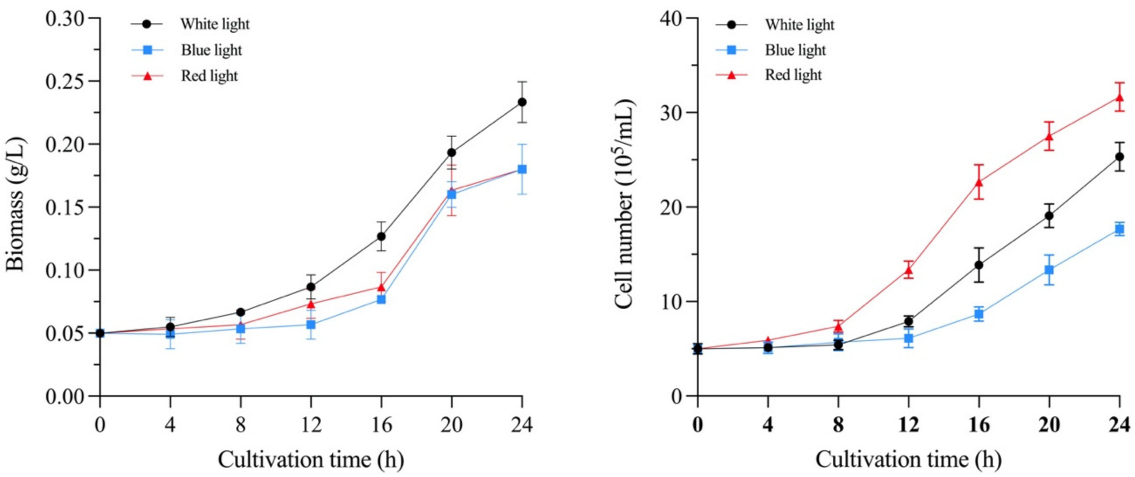

Supplemental Figure S2. Effect of light wavelength on biomass concentration and cell number of *C. reinhardtii* under N-repletion during the first 24 hours. Data were presented in the form of mean ± the standard deviation (*n* = 3).

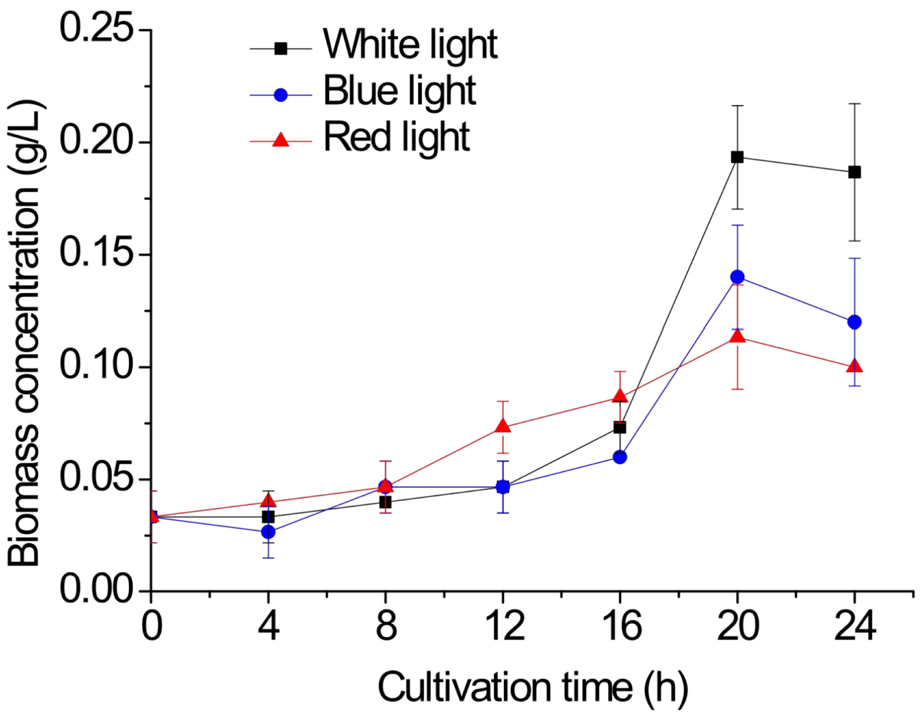

Supplemental Figure S3. Effect of light wavelength on biomass concentration of *C. reinhardtii* under N-recovery. Data were presented in the form of mean ± the standard deviation (*n* = 3).

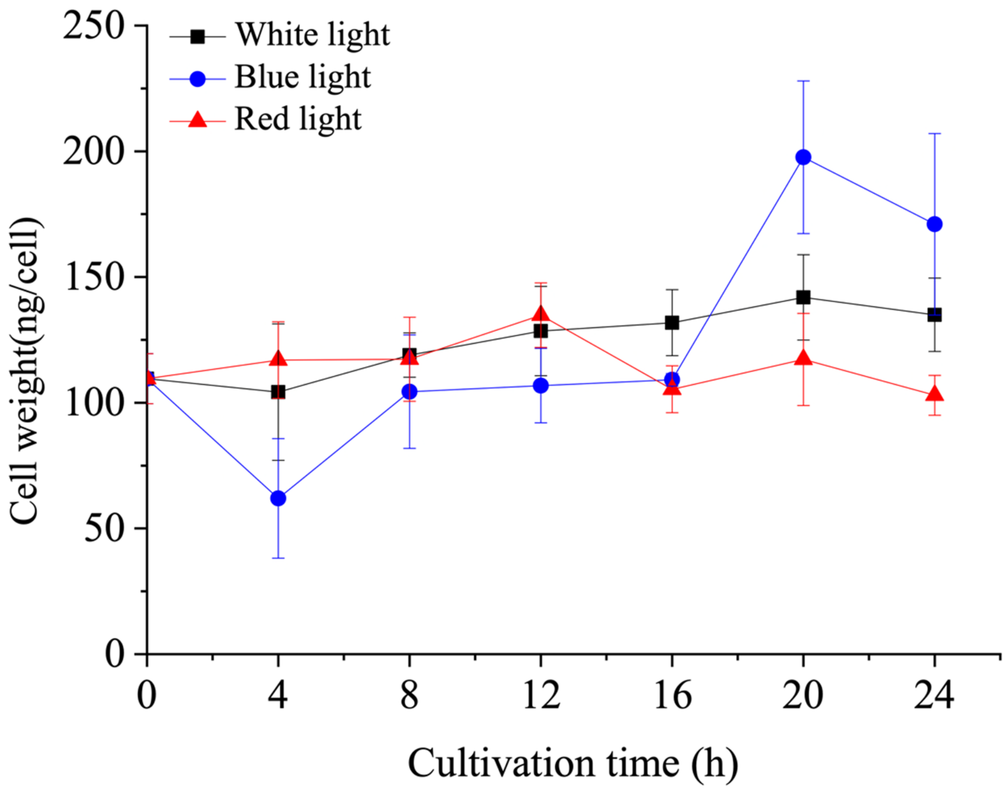

Supplemental Figure S4. Effect of light wavelength on cell weight of *C. reinhardtii* under N-recovery. Data were presented in the form of mean ± the standard deviation (n = 3).

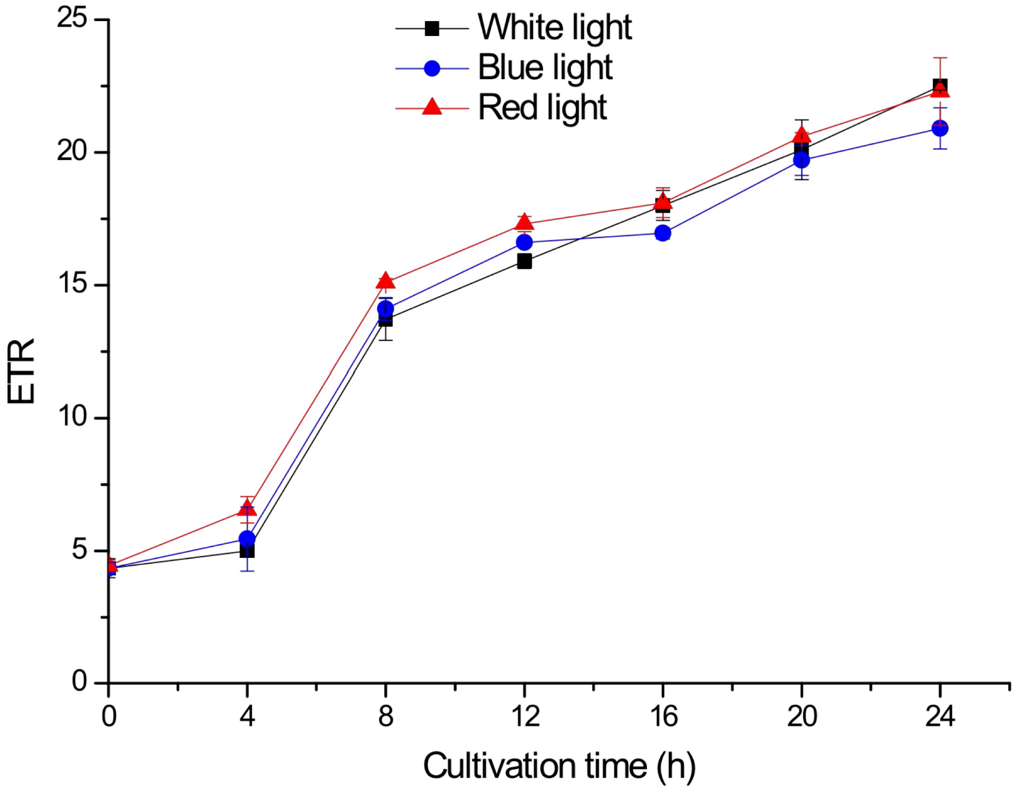

Supplemental Figure S5. Effect of light wavelength on ETR *C. reinhardtii* under N-recovery. Data were presented in the form of mean ± the standard deviation (*n* = 3).

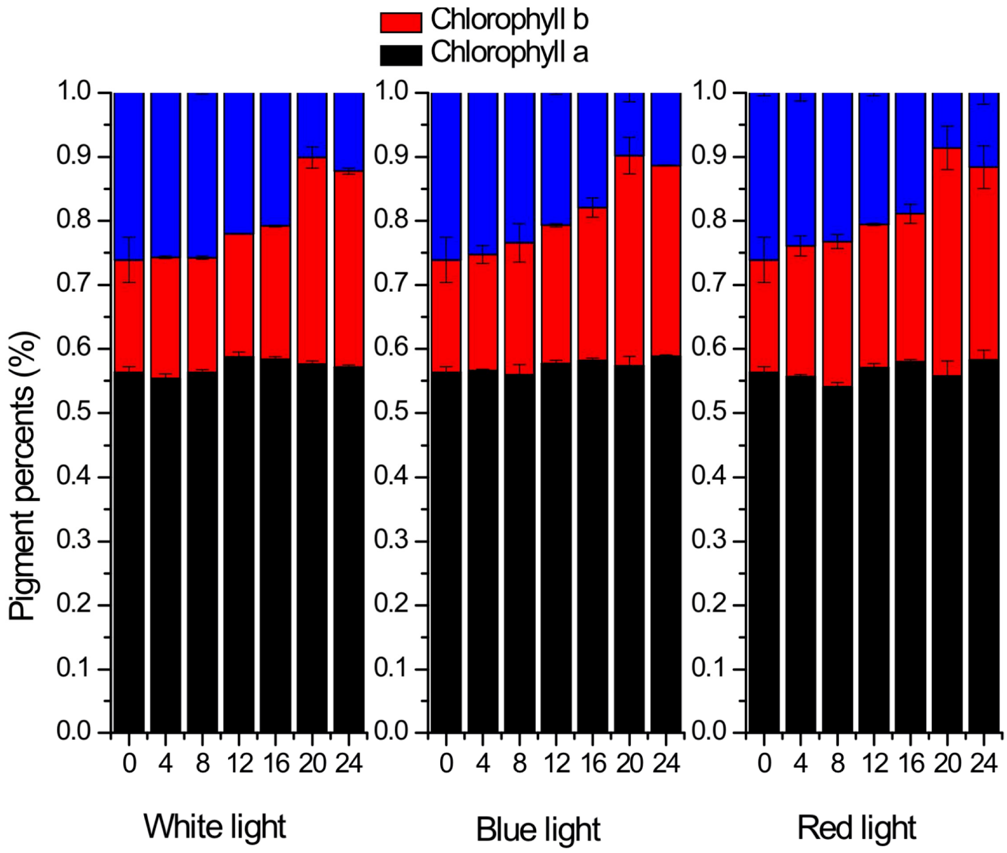

Supplemental Figure S6. Effect of light wavelength on pigment profile of *C. reinhardtii* under N-recovery. Data were presented in the form of mean ± the standard deviation (*n* = 3).

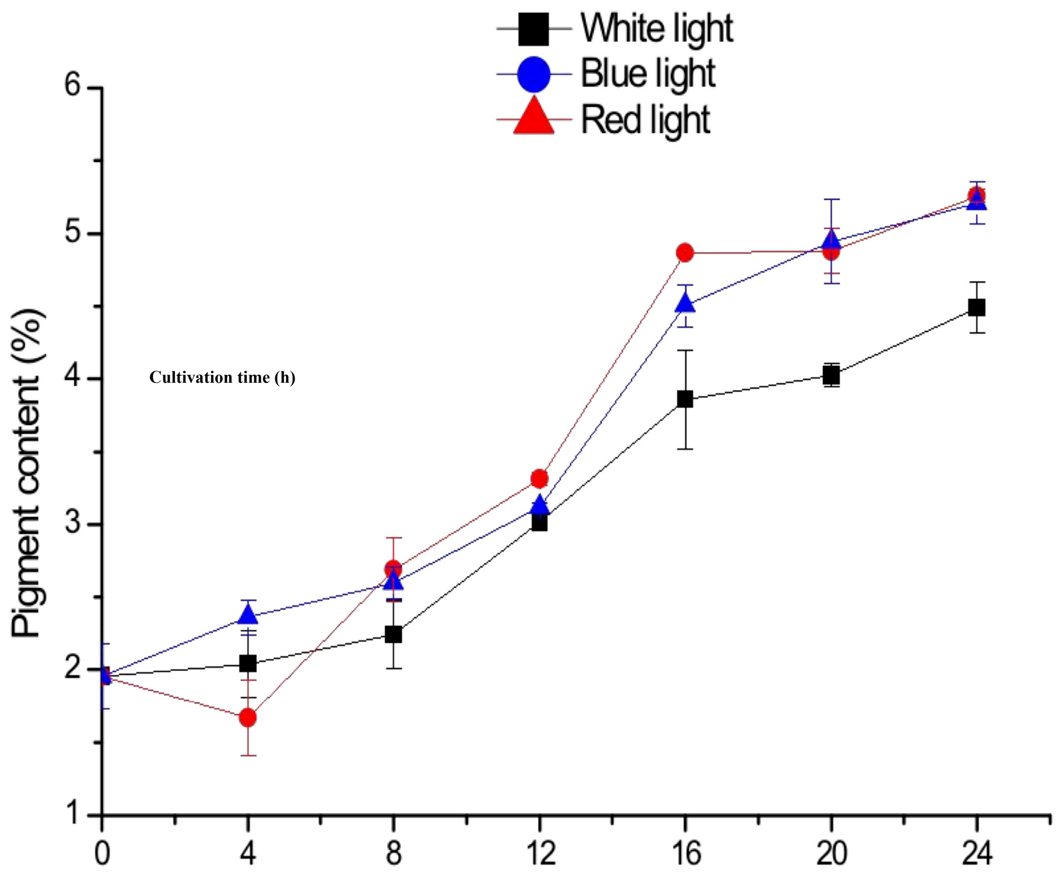

Supplemental Figure S7. Effect of light wavelength on pigment content of *C. reinhardtii* under N-recovery. Data were presented in the form of mean ± the standard deviation (n = 3).

Supplemental Table S1. Fatty acid profiles in TFA of *C. reinhardtii* at different light wavelengths under N-recovery. Data in the table were presented in the form of means (n = 3) ± the standard deviation.

Fatty acid	Fatty acid composition (% TFA)		
	White light	Blue light	Red light
C16:0	27.25 ± 0.86	27.26 ± 0.43	28.56 ± 0.50
C16:1	2.64 ± 0.06	2.57 ± 0.20	N.A.
C16:3	1.69 ± 0.07	1.65 ± 0.02	2.21 ± 0.09
C18:0	7.74 ± 0.06	7.70 ± 0.10	8.49 ± 0.44
C18:1	23.35 ± 0.18	24.04 ± 0.41	24.32 ± 0.34
C18:2	11.58 ±0.23	11.50 ± 0.29	12.44 ± 0.21
C18:3n6	7.31 ± 0.26	6.80 ± 0.52	6.16 ± 0.23
C18:3n3	19.90 ± 0.63	19.48 ± 0.99	17.83 ± 0.07

Data were presented as the percentage of TFA (%);

TFA: total fatty acids;

N.A.: under detectable level;
